# Chlorophyll catabolites in conditioned media of green microalga *Desmodesmus subspicatus*

**DOI:** 10.1007/s10811-015-0618-1

**Published:** 2015-05-20

**Authors:** Krzysztof Grabski, Natalia Baranowski, Joanna Skórko-Glonek, Zbigniew Tukaj

**Affiliations:** Department of Plant Physiology and Biotechnology, Faculty of Biology, University of Gdańsk, Wita Stwosza 59, 80-308 Gdańsk, Poland; Department of Biochemistry, Faculty of Biology, University of Gdańsk, Wita Stwosza 59, 80-308 Gdańsk, Poland

**Keywords:** Green algae, *Desmodesmus*, Conditioned medium, Red pigments, Chlorophyll catabolites

## Abstract

Although the appearance of coloured chlorophyll degradation products of higher plants is well known, knowledge about such compounds produced and released particularly by planktonic algae is still limited. Colourless conditioned media (CM) obtained from autotrophic cultures of unicellular green alga *Desmosdemus subspicatus* turn red after acidification. The accumulation of red pigments in the medium and the growth rate of algae were inversely correlated. The red, crude solution isolated from CM by dialysis and ion exchange chromatography, and next purified by means of high-performance liquid chromatography, appeared to be a mixture of three compounds with characteristic UV/VIS absorption maxima near 330 and 505 nm. Electrospray ionization (ESI) mass spectrometry analysis revealed that the molecular mass of the most polar and most abundant compound was 637 Da and molecular masses of two other ones were 641 and 607 Da. Addition of ^15^ N isotope to the culture medium and subsequent mass spectrometry measurements revealed the occurrence of four nitrogen atoms per each molecule. The data suggest that red pigments isolated from algal-conditioned media are chlorophyll degradation compounds, the production of which depends on light intensity, and are released mainly during the stationary phase of growth.

## Introduction

The degradation path of chlorophyll during developmental processes in higher plants, such as leaf senescence or fruit ripening, is nowadays well elucidated (for a review, see for example Kräutler 2014). It begins with dephytylation followed by the removal of magnesium ion. Finally, oxygenolytic cleavage occurs at position C5 of the porphinoid macrocycle catalyzed by oxygenase (PaO) (Ulrich et al. 2011). As a result, a linear tetrapyrrole is formed—a fluorescent chlorophyll catabolite (pFCC) which is readily transformed into colourless NCC. Nonfluorescent chlorophyll catabolites (NCCs) may spontaneously decompose to rust coloured material, especially in daylight and acidic or oxidizing environment (Bortlik et al. 1990; Moser et al. 2008). In such conditions, dehydrogenation may occur at the C20 meso bridge and at the saturated C15 meso position. As a result, red-shifted absorption is observed with a maximum at around 500 nm.

Contrary to higher plants, not much attention has been paid to degradation of chlorophyll in photosynthetic organisms living in water environments, mainly algae. Phytoplankton converts approximately 50 × 10^15^ g of carbon per year which is close to 40 % of the total amount of carbon dioxide fixed to biomass (after Wilhelm et al. 2004). Algae carry out oxygenic photosynthesis similar to the plants, and the primary pigment absorbing light energy in this process is chlorophyll. Considering that many of the algal species are unicells with a short lifespan, great amounts of chlorophyll degradation products released into water environment from dying or stressed cells could be expected. At present, however, the fate of chlorophyll in planktonic algae is little known. There is a report by Oshio and Hase (1969) that *Chlorella protothecoides*, when grown in nitrogen-deficient but supplemented with organics medium, excretes red pigments to the culture medium which is correlated to the disappearance of chlorophyll from the cells. The structures of these red pigments have been elucidated, and they turned out to be bilin derivatives related to chlorophylls (Engel et al. 1991). Unlike in higher plants, where only pheophorbide *a* is accepted by oxygenase PaO, *Chlorella* red catabolites have been found to originate not only from chlorophyll *a* but also from chlorophyll *b* (Iturraspe et al. 1994). Water-soluble red pigments probably derived from chlorophyll *a* have been isolated also from a Chl *b*-less mutant of *Chlamydomonas reinhardtii* cultured in a medium supplemented with sodium acetate as a carbon source (Doi et al. 1997). To date, no other algal red catabolites have been fully characterized, in contrast to numerous known bile pigments isolated from terrestrial plants (Moser et al. 2009).

In this paper, we present the evidence that red pigments observed in conditioned media of the chlorococcalean green microalga *Desmodesmus subspicatus* are in fact chlorophyll catabolites. These water-soluble components are produced in autotrophic conditions, particularly when cell population reaches the stationary phase of growth.

## Materials and methods

*Desmodesmus subspicatus* (previously *Scenedesmus subspicatus*, Chodat), strain 2594 was obtained from the UTEX collection. The algae were stored on slants aseptically and before each experiment transferred to mineral Bristol’s medium (BBM).

### Cultures and CM preparation

BBM-grown cultures of algae were precultured for several days in 100-mL Erlenmeyer flasks containing 50 mL of suspension. When the suspension reached the exponential growth phase, the material was sampled to set up batch cultures from which CM was prepared. The initial cell density was 0.5 × 10^6^ cells mL^−1^, and the pH of the medium at the start of the culture was adjusted to 6.9 ± 0.1. The cultures were performed in a 3-L flask containing 1.5 L of suspension at 26 °C. The flasks were illuminated from one side with cool white fluorescent tubes (Philips TLD 54 W/94), providing PAR (380–690 nm) of about 160 μmol photons m^−2^ s^−1^ measured at the surface of culture vessels. The cultures were continuously bubbled with air passed through a bacteriological filter (Sartorius 2000, 0.2 μm PTFE). The CM was a spent suspension culture medium harvested 7 days after inoculation. Cells were removed from the medium by centrifugation for 15 min, and supernatant was additionally clarified through membrane filters, 0.8, 0.45 and 0.2 μm (Synpor), applied one by one. The cell-free culture filtrate was a material for further experiments.

### Red pigment isolation and mass spectrometry analysis

Samples of CMs (1000 mL) were evaporated to 50 mL in a rotary evaporator and passed through Amicon Ultra-15 Centrifugal Filter Units (3 kDa). The dialysate obtained (pH 8.5) was applied to a diethylaminoethyl (DEAE) Sephadex A-25 column (bed dimension 3 × 40 cm) previously prewashed with distilled water. When 1 % HCOOH was applied to the column, bounded material turned red and started slowly migrate downwards. The volume of the collected red fraction was around 50 mL. The solution obtained was vacuum dried with a rotary evaporator and further analyzed by high-performance liquid chromatography: Agilent 1100 equipped with an ACE 5 C18 300 column, 250 × 4.6 mm coupled with an Ab Sciex QSTAR XL mass spectrometer. Solvent A was H20 + 0.1 % formic acid, and solvent B was ACN + 0.1 % formic acid. Elution was performed with a linear 10 to 100 % solvent B gradient for 40 min. The flow rate was 1 mL min^−1^. Peak detection was measured at 505 nm. The fractions obtained were analyzed by electrospray ionization–tandem mass spectrometry (ESI–MS/MS) coupled with an HPLC system using a QSTAR XL (Applied Biosystems) in positive-ion mode. The instrument was operated using the following parameters: IS = 5500 V, DP = 60, DP2 = 15, GS1 = 40, CUR = 30, CE = 50.

### Red pigment isotope labelling

*D. subspicatus* cultures were kept in BBM medium with potassium nitrate as the sole nitrogen source. This salt was supplied in an ^15^ N-enriched form (98 atom% K^15^NO_3_, Sigma-Aldrich). Cell culture conditions, CM preparation, red pigment isolation and detection were the same as described above.

### Red pigment secretion

To assess the dynamics of red pigment secretion during the growth of algal suspension, the cultures of *D. subspicatus* were performed in a 3-L flask containing 1.5 L of suspension at 26 °C, 160 μmol photons m^−2^ s^−1^ light intensity, and aerated. Every day, a sample of cell suspension (100 mL) was collected for conditioned media preparation. Each CM was processed in the same manner as described in subchapter “Red pigment isolation and mass spectrometry analysis.” The samples were dialyzed separately, evaporated and loaded onto DEAE resin. Red pigments from the column were eluted with 1 % HCOOH, tenfold concentrated and determined spectrophotometrically at 505 nm.

To assess the effect of light intensity on red pigment excretion, the cultures were kept in a 0.5-L flask containing 0.2 L of suspension at 30 °C and bubbled with atmospheric air. The cultures were illuminated with three light intensities: 200, 80 and 40 μmol photons m^−2^ s^−1^ measured at the surface of culture vessels. After 7 days, 100 mL of each conditioned medium was collected from appropriate culture and was processed in the same manner as described in “Red pigment isolation and mass spectrometry analysis.” The samples obtained were dialyzed, loaded onto DEAE resin, and the red pigment was eluted with 1 % HCOOH, fourfold concentrated and determined spectrophotometrically at 505 nm.

Cell numbers were counted in a Bürker haemocytometer under a light microscope. The growth rate coefficient (*k*) was calculated according to the equation: *k* [day^−1^] = ln *N*_t+1_ − ln *N*_t_ / *t*_t+1_ − *t*_t_, where *N*_*t*+*1*_ and *N*_*t*_ are the numbers of cells at time (days) *t* + 1 and *t*, respectively.

## Results

**Fig. 1:**
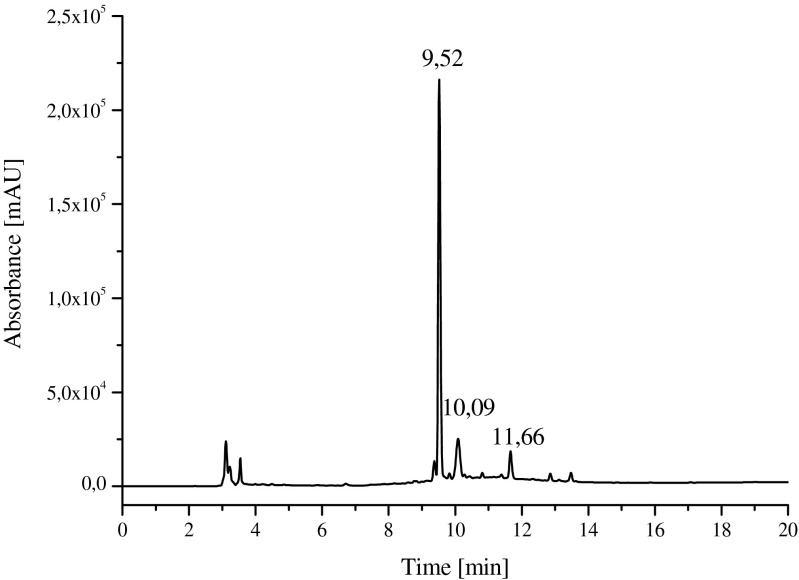
HPLC analysis (recorded at λ = 505 nm) of the red pigments isolated from the conditioned medium of *D. subspicatus* cells

The concentrated yellowish conditioned medium was loaded onto DEAE resin. In the matter of minutes, the bound material became red when exposed to 1 % HCOOH and subsequently red band was eluted from the column with this solvent. The red fraction was collected and further analyzed by reverse-phase chromatography. Figure 1 shows HPLC chromatograms of the crude red pigment solution obtained from *D. subspicatus*. In the conditioned medium from this alga, a few different compounds were detected. The main component which comprises almost all of the red pigments was eluted at 9.5 min, and a few much lower peaks were observed at a retention time of 10.1 and 11.6 min, respectively.

**Fig. 2:**
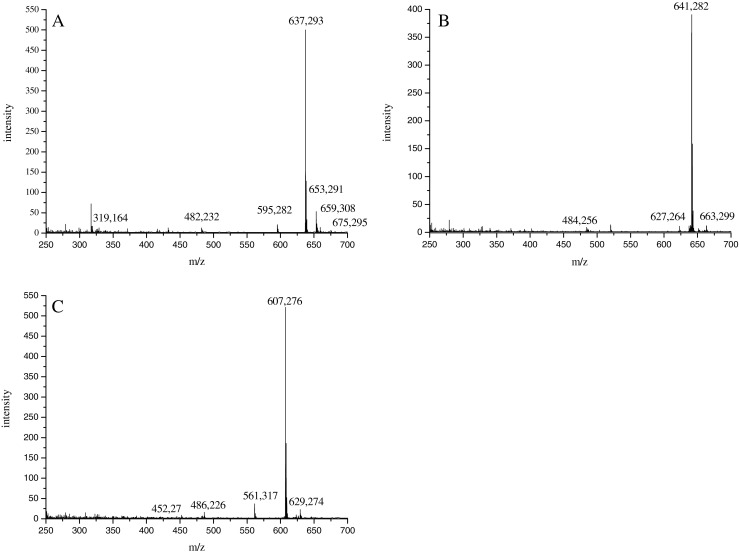
ESI mass spectra in the positive-ion mode of the fractions collected at 9.52 min (spectrum A), 10.09 min (spectrum B) and 11.66 min (spectrum C), isolated from *D. subspicatus*

The molecular masses of all the red pigments were determined in positive-ion mode by ESI mass spectrometry, and the results obtained are depicted in Fig. 2. A singly protonated pseudomolecular ion [M + H]^+^ was observed at *m*/*z* 637.293 (which corresponds to the ion C_33_H_41_N_4_O_9_+, *m*/*z* 637.2868) in HPLC fraction containing the dominant and the most polar compound eluted at 9.5 min, as shown in Fig. 2a. A doubly charged pseudomolecular ion [M + 2H]^2+^ was detected at *m*/*z* 319. An insource fragment ion at *m*/*z* 482 (−155 Da) was also seen in the spectra. Potassiated [M + K]^+^ along with sodiated [M + Na]^+^ adducts were observed at *m*/*z* 675 and 659, respectively. No formation of dimeric species was noted. Figure 2b, c shows pseudomolecular ions of molecules eluted from the C18 column at retention times 10.1 and 11.6 min, respectively. The mass of the second, less polar compound was 640 Da (pseudomolecular ion [M + H]^+^ at *m*/*z* 641.2895 which corresponds to the ion C_32_H_41_N_4_O_10_+, *m*/*z* 641.2817) and of the most hydrophobic molecule 606 Da (ion [M + H]^+^ at *m*/*z* 607.2735 determined as C_32_H_39_N_4_O_8_+, *m*/*z* 607.2762).

**Fig. 3:**
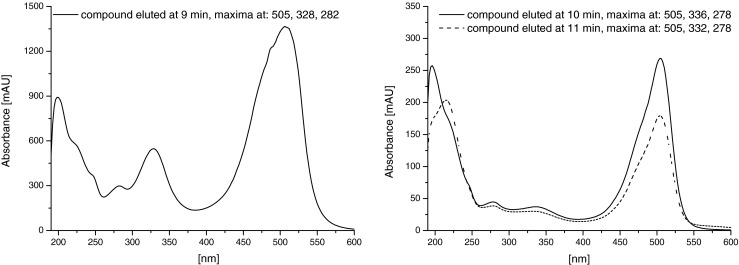
Absorption spectra of compounds from *D. subspicatus* eluted from the C18 column

After evaporation to dryness, the red solid was well soluble in water, methanol, ethanol but not in hydrophobic solvents such as hexane or chloroform (data not shown). The UV/VIS spectra of isolated red pigments are shown in Fig. 3. The spectrum of 637 molecules exhibits two characteristic maxima: near 330 and 505 nm (in water). The other two compounds show strong adsorption only at 505 nm but not at 330.

**Fig. 4:**
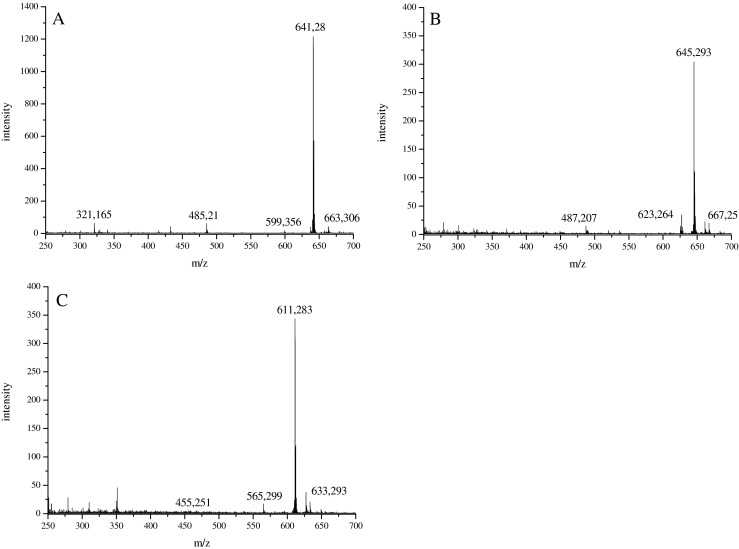
ESI mass spectra in the positive-ion mode of labelled (^15^ N) red pigment fractions collected at 9.52 min (spectrum A), 10.09 min (spectrum B) and 11.66 min (spectrum C), isolated from *D. subspicatus*

HPLC retention times of red pigments labelled with ^15^ N and isolated from *D. subspicatus* were the same as for those unlabelled (data not shown). But, as can be seen in Fig. 4, in all spectra measured mass shifts of 4 Da of all pseudomolecular ions were observed. The mass of the most polar compound was *m*/*z* 641, whereas other masses changed to *m*/*z* 645 and 611, respectively. A doubly charged pseudomolecular ion [M + 2H]^2+^ was detected at *m*/*z* 321, and an insource fragment ion was found at *m*/*z* 485 (−156 Da) in the spectra.

**Fig. 5:**
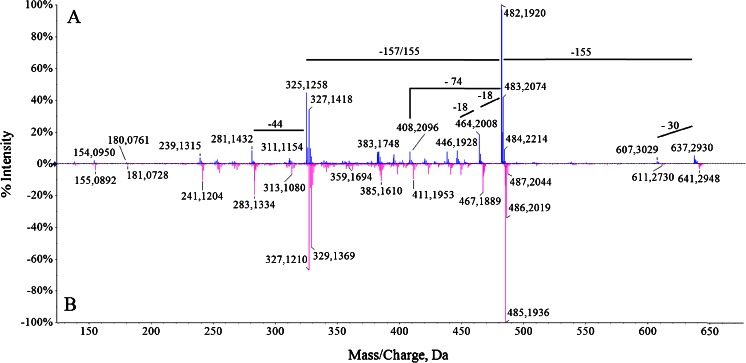
Ion spectra (MS^2^) of *m*/*z* 637: 14 N (**a**) and 15 N (**b**)

**Fig. 6:**
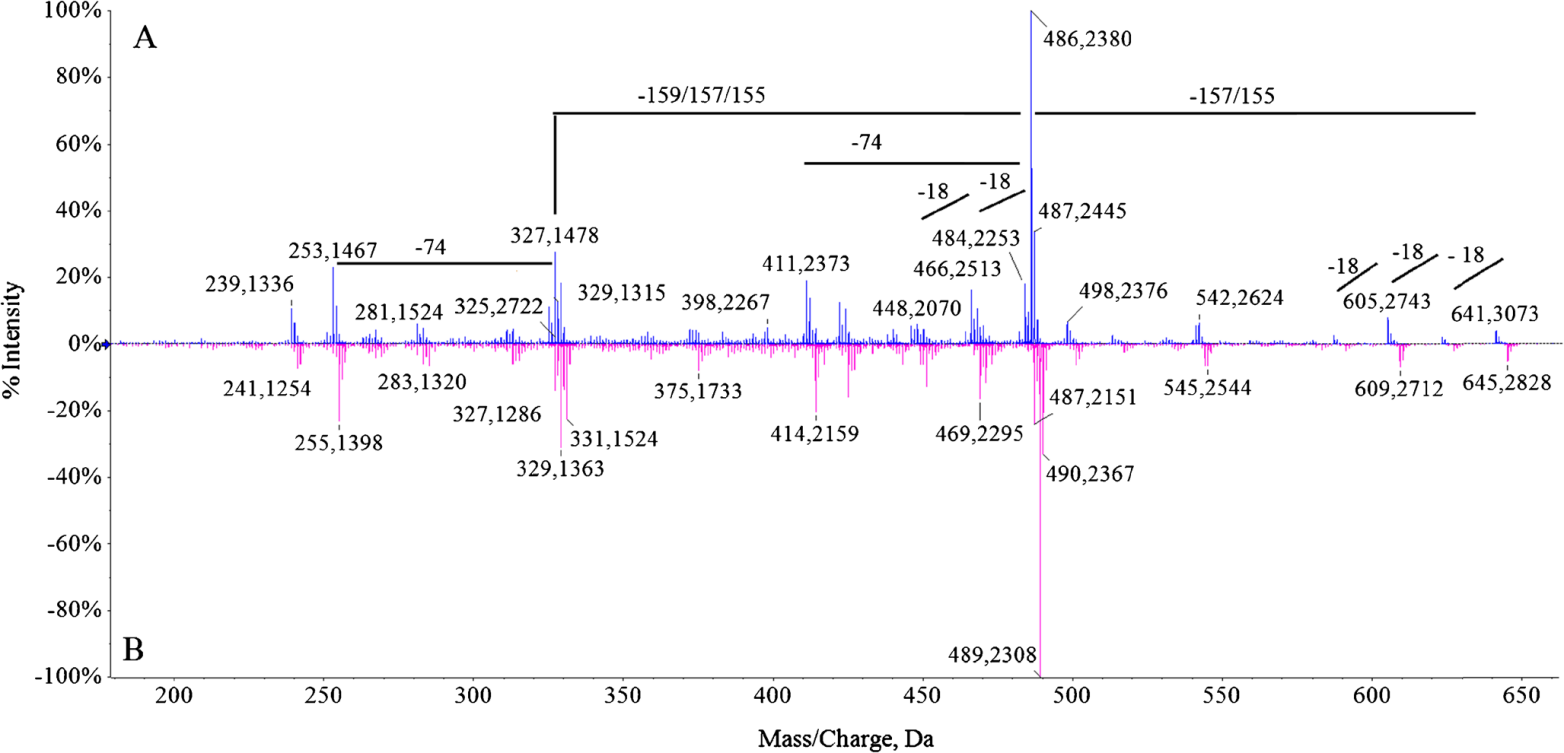
Ion spectra (MS^2^) of *m*/*z* 641: 14 N (**a**) and 15 N (**b**)

**Fig. 7:**
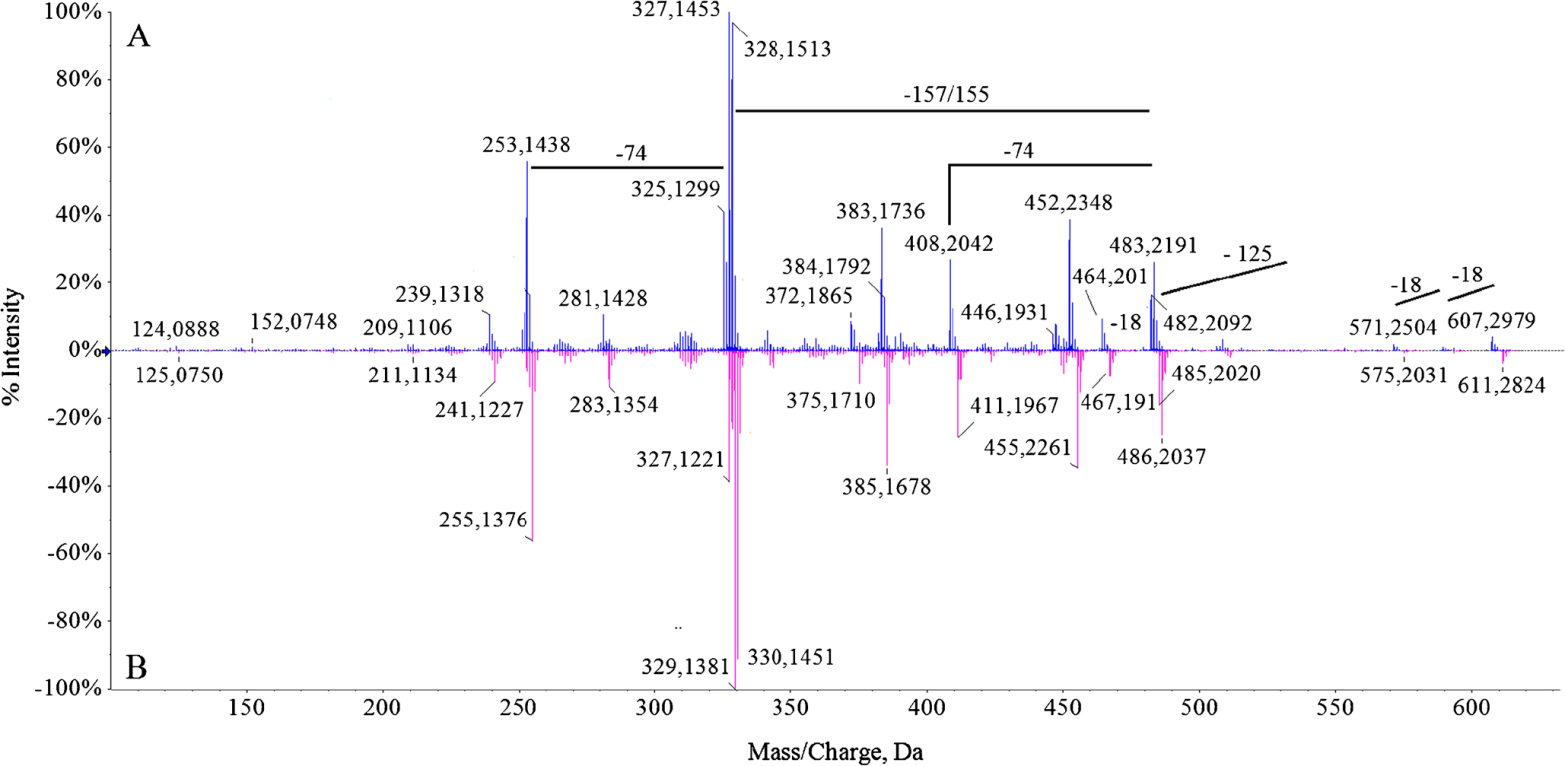
Ion spectra (MS^2^) of *m*/*z* 607: 14 N (**a**) and 15 N (**b**)

The MS^2^ spectra of 637, 641 and 607 (labelled and unlabelled) are depicted in Figs. 5, 6 and 7, respectively. The ion at *m*/*z* 607 originating from parental molecule (*m*/*z* 637) corresponds to loss of 30 Da [M + H–CH_2_O]^+^. The most abundant fragment in MS^2^ spectrum was *m*/*z* 482 [M + H-ring B]^+^. The mass spectrum showed characteristic fragment ion peaks at *m*/*z* 464, 446 and 428 due to multiple losses of H_2_0 probably from the 1,2-dihydroxyethyl group at ring A and propionic acid at ring D. The ions at *m*/*z* 438 and 408 were derived from the peak at *m*/*z* 482 by the loss of CO_2_ and propionic acid, respectively. Signal at *m*/*z* 325 corresponds to loss of another ring [M + H-ring B-ring A]^+^. Figure 5b represents MS^2^ spectrum of 637 ions where all nitrogen atoms were replaced by its heavier counterpart.

The MS^2^ spectra of unlabelled ion *m*/*z* 641 and labelled are shown on Fig. 6a, b, respectively. The most abundant fragment in this MS^2^ spectrum was *m*/*z* 486 [M + H-ring B]^+^. The ion at *m*/*z* 484 might be derived from the precursor ion by the loss of ring A [M + H-ring A]^+^. A few losses of water could be observed from *m*/*z* 641 (*m*/*z* at 623, 605 and 587).

The MS^2^ spectra of unlabelled ion 607 *m*/*z* and labelled are depicted on Fig. 7a, b, respectively. As can be seen, the [M + H]^+^ readily loses water molecules (peaks at *m*/*z* 589 and 571). Fragment ion at *m*/*z* 482 corresponds to loss of ring B and *m*/*z* at 452 to loss of ring B from parental ion. The ion at *m*/*z* 447 and 464 were derived from 482 by the loss of water molecules. Again, *m*/*z* 408 was derived from the peak at *m*/*z* 482 by the loss of propionic acid.

Table 1 shows changes in the cell density, growth rate of the cell population and the accumulation of the red pigment observed during the 8 days of culture. A suspension of *Desmodesmus* cells grown in a batch culture system under autotrophic conditions reached the stationary growth phase after 8 days of culture, when the number of algal cells amounted to about 3 × 10^7^ cells mL^−1^. The concentration of the red pigment in the culture medium increased with the increase in cell density, but the accumulation process and the growth rate of algae were inversely proportional (*r* = −0.747). Rapidly dividing cells (to the third day of culture) released extremely small amounts of red pigments into the medium. The production and releasing suddenly enhanced when the cells almost ceased division at the beginning of the stationary phase of the population growth (sixth day of culture).

**Table 1:** The growth of *Desmodesmus subspicatus* cells and the red pigment content in the conditioned medium

	Light intensity: 160 μmol photons m^−2^ s^−1^			
Time of culture [day]	Number of cells [×10^6^]	*k* [day^−1^]	Absorbance (A) [505 nm]	ΔΑ [*A*_t+1_ − *A*_t_]
1	2.41 ± 0.58	1.57	0.147 ± 0.03	
2	6.75 ± 0.64	1.03	0.209 ± 0.05	0.062
3	12.3 ± 1.55	0.60	0.251 ± 0.06	0.042
4	17.88 ± 0.02	0.37	0.493 ± 0.07	0.242
5	21.6 ± 1.69	0.19	0.707 ± 0.05	0.214
6	26.18 ± 1.38	0.19	1.395 ± 0.12	0.688
7	29.85 ± 0.06	0.13	1.939 ± 0.17	0.544
8	31.31 ± 0.27	0.05	2.921 ± 0.22	0.982

The initial density was 0.5 × 10^6^ cells mL^−1^, “*k*” is the growth rate coefficient, ΔA is the difference between succeeding days of culture (*n* = 4 ± SE)

The effects of light intensity on the secretion of red pigments into CM observed after 7 days of culture are shown in Table 2. Relatively small differences in the final cell density between three experimental variants were accompanied by large differences in amounts of red pigments released into the culture medium. Algae in all variants of 1-week cultures were at different stages of the stationary growth phase. In addition, at the highest light intensity (200 μmol photons m^−2^ s^−1^) cell divisions practically ceased, whereas at 40 μmol photons m^−2^ s^−1^, the density of population still increased. Then, the physiological state of algae determined by population senescence may be more important for the production of red pigments than the direct effect of light intensity. Such an assumption results from the fact that 3.5 times higher absorbances at 505 nm of CM were obtained for a culture grown at the highest light intensity than at the lowest one, whereas cell density of the culture kept at 200 μmol photons m^−2^ s^−1^ was only about 17 % higher than that kept at 40 μmol photons m^−2^ s^−1^.

**Table 2:** The effect of light intensity on cell density and the red pigment content in the 1-week culture spent medium (*n* = 3, ±SE)

	Light intensity [μmol photons m^−2^ s^−1^]		
	200	80	40
Number of cells [×10^6^]	31.15 ± 0.25	28.78 ± 0.29	25.96 ± 0.56
Absorbance [505 nm]	0.415 ± 0.04	0.348 ± 0.06	0.124 ± 0.07

## Discussion

The production of various chlorophyll catabolites (NCC) in many species of higher plants is well documented (Hörtensteiner and Kräutler 2011). It is well-known that these colourless catabolites of the porphyrin moiety can readily decompose to rust-coloured materials within a few minutes when exposed to air, acidic solvents and daylight (Ulrich et al. 2011; Matile et al. 1996). However, little is known about such substances produced and released by algae. So far, only the structures of red chlorophyll catabolites isolated from the culture medium of *C. protothecoides* have been determined (Engel et al. 1991; Iturraspe et al. 1994). Water-soluble red pigments with properties of bile pigments have also been isolated from a chlorophyll *b*-less mutant of *C. reinhardtii*, but their structures were not finally elucidated (Doi et al. 1997). A unique red, open tetrapyrrole structure was isolated from the green macroalga *Bryopsis maxima* with phytol and sugar chain composed of xylose and glucose, but with no aldehyde group at C5 (Miyake et al. 1995). Likewise, the colourless conditioned media studied herein obtained from the green, freshwater alga *D. subspicatus* also readily changed their colour into red in acidic conditions.

ESI mass spectrometry analysis of red pigments secreted by *D. subspicatus* revealed that all their masses are slightly above 600 Da. The mass of the most polar and dominant compound is 637 Da, whereas of the two other 641 and 607 Da. This range of observed masses is in accordance with other known bile pigment masses. The molecular mass of P535 isolated from *C. reinhardtii* and determined by EI mass spectrometry was 605 Da (Doi et al. 1997) whereas for compounds produced by *C. protothecoides* determined by FAB, mass spectrometry amounted to 641, 655 and 597 Da (Iturraspe et al. 1994). Various masses of NCC molecules isolated from *Arabidopsis thaliana* ranged from 600 to 800 Da (Pružinská et al. 2005). *Cercidiphyllum japonicum* is known to produce a molecule with a mass of 629 Da (Oberhuber et al. 2003). A number of incorporated nitrogen atoms are another indicator that molecules produced by *Desmodesmus* cells are related to porphyrins. Chlorophylls and all bile derivatives contain of four nitrogen atoms, one in each ring. Experiments with labelled molecules clearly indicate that all red compounds produced by this alga also contain four nitrogen atoms.

One of the most characteristic features of NCC is their absorption spectrum. These molecules can be nowadays identified in different extracts on the basis of their characteristic UV/Vis absorption properties: because of the α-formyl pyrrole moiety at ring B, they show prominent absorption maxima near 320 nm (Matile et al. 1996). Heme catabolites, another group of common occurring porphyrins, are lacking such formyl group and therefore do not adsorb at 320 nm. The most abundant pigment isolated from conditioned medium tested in this study show a similar absorption maximum near 320 nm. The shifts from 320 nm are probably due to the presence of some substituent at ring B. For example, different location of the hydroxyl group at that ring may change the position of the absorption maximum by a few nanometre (Moser et al. 2009).

The presence of fragment ions in ESI mass spectra is an additional indicator that the observed molecules are derived from chlorophyll. The in-source fragment ion at 482 *m*/*z* originates from parental molecule [M + H]^+^ at 637 *m*/*z*. Such a mass loss (−155 Da) occurs when Chl degradation products are analyzed by soft ionization MS methods and thus is considered to be of a highly diagnostic value (Müller et al. 2014). An odd mass loss may be indicative of a loss of a fragment containing nitrogen. Indeed, the mass shift of the in-source ion fragment from 482 (unlabelled) to 485 (labelled) *m*/*z* suggests that this molecule contains one pyrrole unit less than does [M + H]^+^. In-source fragment ions originating from parental ions at 607 and 641 *m*/*z* are also observed. In addition, the presence on all MS^2^ spectra, only ions that have lost solely one propionate group clearly indicate that all precursor molecules are not heme but chlorophyll catabolites.

To date, chlorophyll conversion to red catabolites in green microalgae was studied in species cultured in heterotrophic conditions. *Chlorella prothothecoides* released red pigments when cells were grown in darkness in a medium with glucose or acetate as a carbon source with simultaneous starvation for nitrogen. (Oshio and Hase 1969). Also, a mutant *C. reinhardtii* excretes red pigments into the culture medium during growth in complete darkness on acetate as a carbon source (Doi et al. 1997). *D. subspicatus* cells can release products of chlorophyll degradation when cells are grown in autotrophic conditions. The secretion rapidly increased when the cell population reached the stationary phase and algal suspension became yellowish but without colouration of the medium. We previously observed that the decreasing rate of cell division and, in consequence, a culture senescence was due to nitrogen depletion (Grabski and Tukaj 2008). Under N-starvation, different species of algae undergo degreening, inhibition of photosynthetic oxygen evolution or increased carotenogenesis etc. (Sayed 1998; Berges and Falkowski 1998). Here, nitrogen shortage could be an important factor indirectly inducing chlorophyll degradation in *Desmodesmus* cells. Similarly, we suppose that light intensity, crucial for autotrophic growth of cells, also indirectly affects red pigment production, but the effect of light, nutrients or stress conditions on the production and particularly excretion of chlorophyll catabolites needs more detailed studies.

## References

[CR1] Berges AJ, Falkowski GP (1998). Physiological stress and cell death in marine phytoplankton: induction of proteases in response to nitrogen or light limitation. Limnol Oceanogr.

[CR2] Bortlik K-H, Peisker C, Matile P (1990). A novel type of chlorophyll catabolite in senescent barley leaves. J Plant Physiol.

[CR3] Doi M, Shima S, Egashira T, Nakamura K, Okayama S (1997) New bile pigment excreted by a *Chlamydomonas reinhardtii* mutant: a possible breakdown catabolite of chlorophyll *a*. J Plant Physiol 150:504–508

[CR4] Engel N, Jenny TA, Mooser V, Gossauer A (1991). Chlorophyll catabolism in *Chlorella protothecoides*. Isolation and structure elucidation of a red bilin derivative. FEBS Lett.

[CR5] Grabski K, Tukaj Z (2008) Autoinduction activity of a conditioned medium obtained from high density cultures of the green alga *Scenedesmus subspicatus*. J Appl Phycol 20:323–330

[CR6] Hörtensteiner S, Kräutler B (2011). Chlorophyll breakdown in higher plants. BBA-Bioenergetics.

[CR7] Iturraspe J, Engel N, Gossauer A (1994) Chlorophyll catabolism. Isolation and structure elucidation of chlorophyll *b* catabolites in *Chlorella protothecoides*. Phytochemistry 35:1387–1390

[CR8] Kräutler B (2014). Phyllobilins—the abundant bilin-type tetrapyrrolic catabolites of the green plant pigment chlorophyll. Chem Soc Rev.

[CR9] Matile P, Hortensteiner S, Thomas H, Kräutler B (1996). Chlorophyll breakdown in senescent leaves. Plant Physiol.

[CR10] Miyake K, Ohtomi M, Yoshizawa H, Sakamoto Y, Nakayama K, Okada M (1995). Water soluble pigments containing xylose and glucose in gametangia of the green alga, *Bryopsis maxima*. Plant Cell Physiol.

[CR11] Moser S, Ulrich M, Müller T, Kräutler B (2008). A yellow chlorophyll catabolite is a pigment of the fall colours. Photochem Photobiol Sci.

[CR12] Moser S, Müller T, Oberhuber M, Kräutler B (2009). Chlorophyll catabolites—chemical and structural footprints of a fascinating biological phenomenon. Eur J Org Chem.

[CR13] Müller T, Vergeiner S, Kräutler B (2014). Structure elucidation of chlorophyll catabolites (phyllobilins) by ESI-mass spectrometry. Pseudo-molecular ions and fragmentation analysis of a nonfluorescent chlorophyll catabolite (NCC). Int J Mass Spectrom.

[CR14] Oberhuber M, Berghold J, Breuker K, Hortensteiner S, Kräutler B (2003). Breakdown of chlorophyll: a nonenzymatic reaction accounts for the formation of the colorless “nonfluorescent” chlorophyll catabolites. Proc Nal Acad Sci.

[CR15] Oshio Y, Hase E (1969). Studies on red pigments excreted by cells of Chlorella protothecoides during the process of bleaching induced by glucose or acetate. I. Chemical properties of the red pigments. Plant Cell Physiol.

[CR16] Pružinská A, Tanner G, Aubry S, Anders I, Moser S, Müller T, Ongania K-H, Kräutler B, Youn J-Y, Liljegren JS, Hortensteiner S (2005). Chlorophyll breakdown in senescent *Arabidopsis* leaves. Characterization of chlorophyll catabolites and of chlorophyll catabolic enzymes involved in the degreening reaction. Plant Physiol.

[CR17] Sayed HO (1998). Analysis of photosynthetic responses and adaptation to nitrogen starvation in *Chlorella* using in vivo chlorophyll fluorescence. Photosynthetica.

[CR18] Ulrich M, Moser S, Müller T, Kräutler B (2011). How the colourless nonfluorescent chlorophyll catabolites rust. Chem Eur J.

[CR19] Wilhelm C, Becker A, Toepel J, Vieler A, Rautenberger R (2004). Photophysiology and primary production of phytoplankton in freshwater. Physiol Plant.

